# Application of Molecular Vapour Deposited Al_2_O_3_ for Graphene-Based Biosensor Passivation and Improvements in Graphene Device Homogeneity

**DOI:** 10.3390/nano11082121

**Published:** 2021-08-20

**Authors:** Muhammad Munem Ali, Jacob John Mitchell, Gregory Burwell, Klaudia Rejnhard, Cerys Anne Jenkins, Ehsaneh Daghigh Ahmadi, Sanjiv Sharma, Owen James Guy

**Affiliations:** 1 Centre for NanoHealth, College of Engineering, Swansea University, Swansea SA2 8PP, UK; j.j.mitchell@swansea.ac.uk (J.J.M.); e.daghighahmadi@swansea.ac.uk (E.D.A.); 2 Department of Physics, College of Science, Swansea University, Swansea SA2 8PP, UK; g.burwell@swansea.ac.uk (G.B.); 988211@swansea.ac.uk (K.R.); 3 School of Medicine, Swansea University, Swansea SA2 8PP, UK; cerys.jenkins@swansea.ac.uk; 4 Faculty of Science and Engineering, Bay Campus, Swansea University, Swansea SA1 8EN, UK; sanjiv.sharma@swansea.ac.uk; 5 Department of Chemistry, College of Science, Swansea University, Swansea SA2 8PP, UK

**Keywords:** graphene, passivation, molecular vapour deposition, biosensors, aluminium oxide

## Abstract

Graphene-based point-of-care (PoC) and chemical sensors can be fabricated using photolithographic processes at wafer-scale. However, these approaches are known to leave polymer residues on the graphene surface, which are difficult to remove completely. In addition, graphene growth and transfer processes can introduce defects into the graphene layer. Both defects and resist contamination can affect the homogeneity of graphene-based PoC sensors, leading to inconsistent device performance and unreliable sensing. Sensor reliability is also affected by the harsh chemical environments used for chemical functionalisation of graphene PoC sensors, which can degrade parts of the sensor device. Therefore, a reliable, wafer-scale method of passivation, which isolates the graphene from the rest of the device, protecting the less robust device features from any aggressive chemicals, must be devised. This work covers the application of molecular vapour deposition technology to create a dielectric passivation film that protects graphene-based biosensing devices from harsh chemicals. We utilise a previously reported “healing effect” of Al_2_O_3_ on graphene to reduce photoresist residue from the graphene surface and reduce the prevalence of graphene defects to improve graphene device homogeneity. The improvement in device consistency allows for more reliable, homogeneous graphene devices, that can be fabricated at wafer-scale for sensing and biosensing applications.

## 1. Introduction

The demand for point-of-care (PoC) devices is increasing, with the identification of new pathogens and increased testing for infectious diseases, such as COVID-19 [1,2]. Standard diagnostic laboratory tests require expensive equipment and trained professionals which takes time for processing, whereas PoC tests are rapid and cheaper, providing results in a matter of minutes in a clinical or remote setting. These low-cost devices are portable, easy to use and store, and do not require intervention by trained professionals.

Discovered in 2004 by Andre Geim and Konstatin Novoselov, graphene is a near-transparent two-dimensional (2D) structure that consists of hybridised sp^2^ carbon atoms, arranged in a flat honeycomb-like lattice [3]. It is one of the strongest materials ever discovered, with an ultimate tensile strength of 125 GPa [4,5,6], and impressive anti-corrosive properties [7]. Graphene is incredibly versatile and heavily researched in various fields, such as chemical sensors [8], biosensors [9,10,11,12,13,14,15], high-speed transistors [16], supercapacitors [17], solar panel cells [18], battery technology [19], and point-of-care (PoC) devices [20,21]. Due to its excellent electron mobility and high electron transfer rate, graphene is advantageous for biosensors and PoC sensing devices [3,5,22]. Work has already been performed on graphene biosensing devices for hepatitis B detection [9] and the early detection of cancer risk [12,13]. Bioreceptors, such as antibodies, can be bound to the graphene surface, for real-time virus detection [23]. There are numerous chemical functionalisation approaches available for the modification of the graphene surface for biosensing applications. These include π-π stacking of molecules with functional groups which assist biosensor coupling, hydrogen bond interactions or direct covalent attachment to the graphene [24,25,26]. Non-covalent methods of graphene functionalisation do not alter the graphene crystalline structure, allowing for the graphene to maintain its high electron mobility [27]. Covalent methods of chemical functionalisation convert the sp^2^ carbon-carbon bonds in the graphene to sp^3^ bonds and can be achieved using various functionalisation methods, such as diazotization [28,29]. The functionalisation chemistries available for graphene are extremely versatile as the functionalised molecules can be used for sensing or can act as an intermediary for biomolecular attachment [14,30].

However, whilst graphene is chemically robust, other elements of graphene biosensors, for example, the metal electrode that interconnects to the active graphene layer, can be degraded by the functionalization chemistry or by exposure to the test analyte fluids. These chemical interactions can interfere with the electrical and electrochemical transduction in the graphene sensor device and affect device reliability. Therefore, it is desirable to confine exposure to chemical interactions to the active graphene component of the device only, during solution-based functionalisation processes or biosensing measurements that occur in the solution. This confinement can be achieved using a passivation layer to provide a protective barrier between any liquid media and the metal electrodes, whilst allowing full contact with the graphene [31]. Previous reports have shown successful applications of passivation for graphene biosensing applications. Walters et al. utilised screen-printed dielectric ink to coat graphene resistor devices, protecting the metal tracks in their device and exposing the graphene for sensing [9]. Other graphene biosensing devices have been fabricated, which utilise ALD aluminium oxide (Al_2_O_3_) deposition directly on top of the graphene [31,32]. These protective layers must be chemically inert to most acids and solvents, and stable under atmospheric conditions to provide adequate protection. For biosensing applications, it is imperative that there is a window in the passivation layer which allows the graphene surface to be exposed for effective functionalisation and subsequent biosensing.

High-κ dielectrics are commonly used for passivation layers in semiconductor devices [33,34,35] and solar cells [36]. Dielectrics are effective passivation materials as they are electrically and thermally insulating and can be easily scaled up for wafer-scale fabrication. There are many methods of depositing dielectrics onto graphene, such as evaporation [37,38], sputtering [39], and atomic layer deposition (ALD) [40]. Physical vapor deposition (PVD) processes, such as magnetron sputtering deposition, and plasma-enhanced chemical vapour deposition (PECVD) can damage graphene devices, forming defects and reducing the carrier mobility [39,41,42]. ALD is a less destructive deposition technique that uses precursor materials, that are sequentially injected into a deposition chamber, to promote the growth of uniform and conformal materials. It creates much higher quality high-k dielectrics than either PVD or CVD, with better thickness control and less damage to the graphene, but is much slower than sputtering or evaporation [43,44]. ALD deposition commonly require standard operating temperatures above 100 °C which can reduce compatibility with certain substrates, such as metalised substrates or thin, flexible polymers [40,43,45,46]. However, low-temperature ALD deposition processes have also been previously reported [47]. Molecular vapour deposition (MVD) is a batch process tool from SPTS Technologies, which utilises both ALD and MLD technologies to create high quality uniform inorganic and organic coatings, such as metal oxides and self-assembled monolayers (SAMs) [48]. One benefit of MVD, over other thin-film deposition processes, is that the standard operating temperature of MVD is lower, with processes capable of running at 100 °C and below. This increases compatibility with more materials whilst using fewer precursor resources and reducing overall usage costs whilst maintaining film quality.

In this study, we report the development of an Al_2_O_3_ passivation layer, deposited using molecular vapour deposition, for use in graphene sensors and biosensors. Following the Al_2_O_3_ deposition on graphene, the Al_2_O_3_ etch rate was optimised to allow for controlled etching with minimal photoresist mask degradation. Graphene surfaces of passivated and non-passivated graphene devices were electrochemically functionalised with poly(1,5-diaminonaphthalene) (pDAN). The condition of the metal tracks in both devices were then characterised. ALD Al_2_O_3_ passivation processes have also been reported to reduce organic polymer contaminants present on the graphene surface, related to photolithographic processing [49]. In this work, spectroscopic characterisation techniques have been used to confirm the reduction of the surface organic impurities when using the MVD Al_2_O_3_ passivation layer. Al_2_O_3_ passivation using MVD is shown to produce reproducible graphene biosensors for fast and reliable point-of-care diagnostics in liquid media.

## 2. Materials and Methods

### 2.1. Materials

Chemical vapour deposited (CVD) monolayer graphene grown on Cu substrate and transferred on 300 nm thermal SiO_2_/525 μm Si wafers using PMMA-based wet transfer were supplied by Graphenea Inc (San Sebastián, Spain) [50]. Microposit LOR 3A Photoresist and Microposit S1805 G2 positive photoresist were supplied by DOW Electronics Materials (Marlborough, MA, USA). The 2″ diameter chromium (Cr) and palladium (Pd) PVD targets were obtained from Kurt J Lesker (St. Leonards-on-Sea, East Sussex, UK). TechniStrip NI555, AZ nLof 2070 photoresist, AZ 726 MIF Developer, and 25% TMAH etchant were supplied by MicroChemicals GmbH (Ulm, Germany). Trimethylaluminium (TMA) precursor is supplied by Pegasus Chemicals Ltd. (Sandycroft, UK). The 1,5-diaminonaphthalene (DAN) and 95–98% sulfuric acid (H_2_SO_4_) solution was supplied by Sigma-Aldrich (Gillingham, Dorset, UK). Type II DI water with the ASTM D1193 standard and a resistance of 18 MΩ.cm was produced using a Merck Millipore Elix 3 water purification system (Darmstadt, Germany).

### 2.2. Characterisation Methods

The Al_2_O_3_ film thickness was measured using a J.A. Woollam M-2000 spectroscopic ellipsometer (Lincoln, NE, USA) at various locations at a 65–75° angle, in steps of 5°. Once measured, using CompleteEASE software (version 5.23, J.A. Woollam, Lincoln, NE, USA), the data are fitted with a Cauchy model. The surface morphology was characterised using a Keyence VHX-950F Series microscope (Milton Keynes, UK), a Hitachi S4800 SEM (Maidenhead, UK), and a JPK NanoWizard II AFM (Berlin, Germany). SEM and EDX images were taken on the Hitachi S4800 (Maidenhead, UK) with an acceleration of 10 kV and an emission current of 10 μA. The AC tapping mode atomic force microscopy (AFM) images were taken using NCHV AFM probes, supplied by Bruker Ltd. (Coventry, UK), with a resonant frequency, spring constant, and tip radius of 320 kHz, 40 N/m, and 8 nm, respectively. The AFM data were analysed using Gwyddion software (version 2.57, Czech Metrology Institute, Jihlava, Czechia). Optical microscope images were taken with a Keyence VHX-950F Series Microscope (Milton Keynes, UK). Raman spectra were acquired using a Renishaw Qontor inVia Raman microscope (Wotton-under-Edge, UK) with a 532 nm laser at 10 mW with a ×100 objective lens, and the data were analysed using a custom MATLAB script (version R2020b, MathWorks, Portola Valley, CA, USA). X-ray photoelectron spectroscopy (XPS) was performed using a Kratos Axis Supra XPS (Manchester, UK) with an Al Kα monochromatic source, with an emission current of 15 mA and a pass energy of 20 eV. Where applicable, adventitious carbon was etched off the surface using a 5 keV Ar gas cluster ion source (GCIS) at 45 nA for 100 s during the XPS measurement. Each sample had a minimum of three measurements performed to ensure that the resultant scans were an accurate representation of the graphene surface. Data analyses from XPS measurements were performed using CasaXPS software (version 2.3.23rev1.1K, Casa Software Ltd.).

### 2.3. Graphene Resistive Sensor Device Fabrication and MVD Passivation Layer Process

**Figure 1:**
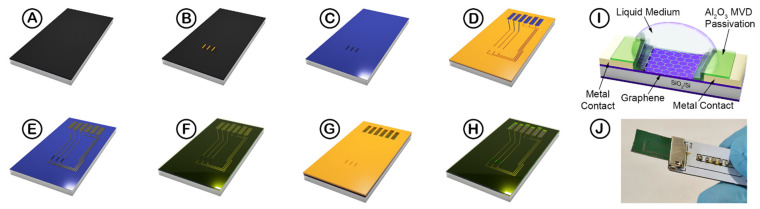
Graphene resistive sensor device fabrication process schematic. (**A**) A graphene-on-SiO_2_/Si substrate; (**B**) application of the photoresist etch mask; (**C**) after etching the graphene with O_2_ plasma and subsequent photoresist mask removal; (**D**) coating and patterning photoresist for metal electrodes; (**E**) after depositing 30 nm Cr/200 nm Pd and lift-off procedure; (**F**) depositing 50 nm Al_2_O_3_ with MVD deposition technique; followed by (**G**) creating the negative resist etch mask to selectively etch the Al_2_O_3_ to expose the graphene and metal contacts; (**H**) Al_2_O_3_ is etched with 1.25% TMAH and the photoresist mask is removed; (**I**) cross-section of the passivated graphene resistor device with liquid media contacting the graphene, and (**J**) passivated graphene resistive sensor device inserted into a custom connector.

**Figure 2:**
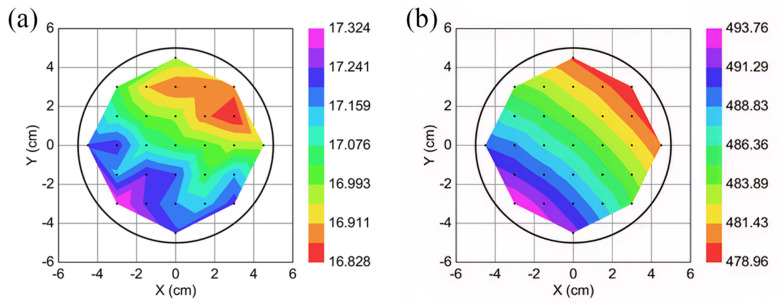
Ellipsometry map measurements of MVD Al_2_O_3_-on-Si wafers, showing the thickness (in Å) across the wafer. (**a**) The 29-point ellipsometry measurement of a bare 4″ Si wafer with 1.7 nm native oxide before Al_2_O_3_ MVD deposition; (**b**) the 29-point ellipsometry measurement of a 4″ Si wafer after 50 nm Al_2_O_3_ MVD deposition.

The CVD graphene wafer was annealed at 550 °C for 10 min under a vacuum with 50 sccm Ar flow, using a Jiplec Jetfirst 200 Rapid Thermal Annealing system (ECM USA Inc., Pleasant Prairie, WI, USA) before photolithographic patterning using a bi-layer resist process (LOR 3A/S1805) (Figure 1B), by the same parameters as reported in our previous work [9]. Once the photoresist had been patterned, the exposed graphene was then etched with O_2_ plasma, at 80 W power, and 7 × 10^−1^ mBar pressure, for 5 min using a Quorum Emitech K1050X RF Plasma Asher (Quorum Technologies Ltd., Sussex, UK), before the remaining photoresist (used to protect the graphene during plasma etching) was removed by submerging the wafer in DMSO, warmed to 80 °C, for 1 h, revealing the graphene channels (Figure 1C). Once the photoresist was removed, a lithography process using the same resist was reapplied, to expose the ends of the graphene channels to the stacked 30 nm Cr/200 nm Pd metal contacts (Figure 1D). The Cr/Pd metal stack was deposited using magnetron sputtering in a Kurt J Lesker PVD75 system (St. Leonards-on-Sea, East Sussex, UK). Following metal deposition, the photoresist was removed using a photoresist lift-off process with DMSO at 80 °C, for 2 h (Figure 1E). Once lift-off was completed, the graphene devices were coated with a ~50 nm layer of Al_2_O_3_ deposited at 100 °C using TMA and water vapour precursors (Figure 1F) using an SPTS MVD300 system. The pressures used for the TMA and water vapour are 1.3 and 1.0 torr, respectively. With a blank Si wafer, the Al_2_O_3_ thickness and uniformity, before and after MVD deposition, was characterised using ellipsometry (Figure 2).

After MVD deposition, a negative photoresist etch mask was patterned, exposing windows for the graphene and device metal contacts (Figure 1G). Once the photoresist mask is patterned, the devices were immersed in tetramethylammonium hydroxide (TMAH) etchant to remove the exposed Al_2_O_3_. The optimised TMAH concentration and resulting etch rate are discussed later in this study. Once the Al_2_O_3_ is etched, with the graphene exposed, the photoresist mask was subsequently removed by immersing the wafer in NI555 resist remover at 80 °C for 1 h, revealing the graphene devices (Figure 1H). A cross-section of the graphene device can be seen in Figure 1I. Once the graphene sensors are fabricated, the wafers were diced so that the devices can fit into a custom electrical connector, supplied by Biovici Ltd. (Swansea, UK) (Figure 1J), described in the previous work [9].

### 2.4. The pDAN Functionalisation Process

The graphene channels in the devices are modified by electrochemically functionalising the surface with DAN, electropolymerising it to form pDAN. These processes were performed using an Autolab PGSTAT302N (Metrohm Ltd., Tetbury, UK) in a standard three-electrode cell. The pDAN functionalisation process utilises 0.25 M sulfuric acid (H_2_SO_4_) and 10 mM DAN in a three-electrode system, with an Ag/AgCl reference electrode and a Pt wire serving as the counter electrode. The working electrode is the graphene chip, with the graphene immersed in the H_2_SO_4_/DAN solution. After setting up the three-electrode system, cyclic voltammetry (CV) scans were performed for five cycles at 50 mV/s from −0.9 to 0.6 V.

## 3. Results and Discussion

### 3.1. Aluminium Oxide (Al_2_O_3_) TMAH Etch Optimisation

For sensing applications, the sensor element must be exposed through a “window” in the passivation layer. Al_2_O_3_ can be etched to form a window in the layer using a variety of dry [51] and wet [52] chemical etching methods. Dry etch methods, have a risk of etching through the Al_2_O_3_ layer and damaging the graphene channels underneath [53]. Therefore, wet etching is the preferred method to remove the Al_2_O_3_. Al_2_O_3_ can be removed in either highly concentrated acids or bases [52,54]. However, utilising highly concentrated acids will likely result in oxidising the graphene, reducing the graphene’s electrical conductivity [55,56,57]. TMAH is a strong base that has been used to etch aluminium [54] and can also be used to etch away Al_2_O_3_ [58].

**Figure 3:**
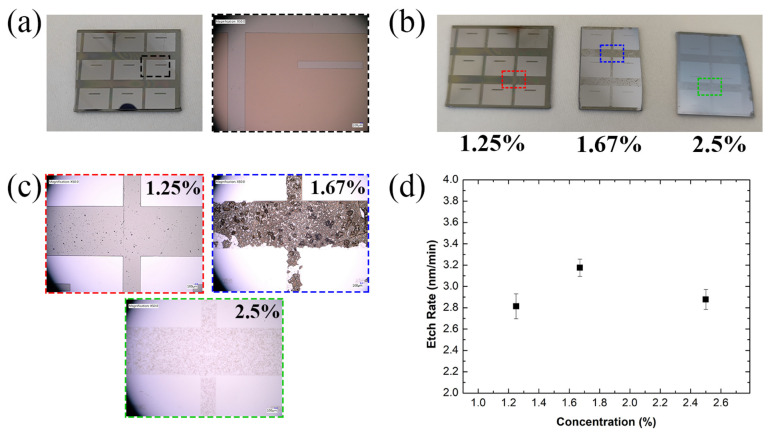
Al_2_O_3_-coated Si pieces that are coated and patterned with an nLof 2070 photoresist mask during TMAH optimisation etch tests. (**a**) Al_2_O_3_-coated Si pieces before etching, with a zoomed-in optical microscope image of the area in a black box. (**b**) Al_2_O_3_-coated Si pieces after 15 min of TMAH etching, at different concentrations, with (**c**) zoomed-in optical microscope images displaying the condition of the photoresist mask, each correlating with the coloured boxes found in Figure 3b. (**d**) Average etch rate of Al_2_O_3_ in TMAH at 21 °C, with respect to the TMAH concentration.

For wet chemical etching, AZ nLof 2070 was used as the photoresist mask as it can provide a thick resist layer for satisfactory selective protection of areas of the Al_2_O_3_ layer. Since photoresist is partially soluble in TMAH, an optimised concentration was required to greatly reduce the etch rate of the photoresist mask. In addition, 25% TMAH was diluted down with DI water to concentrations of 1.25, 1.67, and 2.5%. Al_2_O_3_-coated Si pieces were measured using ellipsometry before being patterned with nLof 2070 photoresist. The samples were immersed in different TMAH concentrations for a total of 15 min at room temperature (21 °C) and were measured periodically via ellipsometry to determine the overall etch rate (Figure 3). A majority of the photoresist masks were dissolved after being immersed in the 2.5 and 167% TMAH etchants. However, 1.25% TMAH had a minimal effect on the photoresist mask and was intact. The etch rate was roughly similar amongst all the tested TMAH concentrations, with a small variation due to error, and 1.25% TMAH having an etch rate of 2.81 nm/min. This is due to the fact that the etch rate is proportional to the etchant temperature and the TMAH concentration has little effect on Al_2_O_3_ etching at 21 °C [58,59,60]. Therefore, lower concentrations of TMAH can be used to etch Al_2_O_3_ without degrading the photoresist mask, whilst maintaining the same etch rate.

### 3.2. Graphene Device Characterisation

#### 3.2.1. Surface Morphology—SEM and Energy-Dispersive X-ray Spectroscopy (EDX)

**Figure 4:**
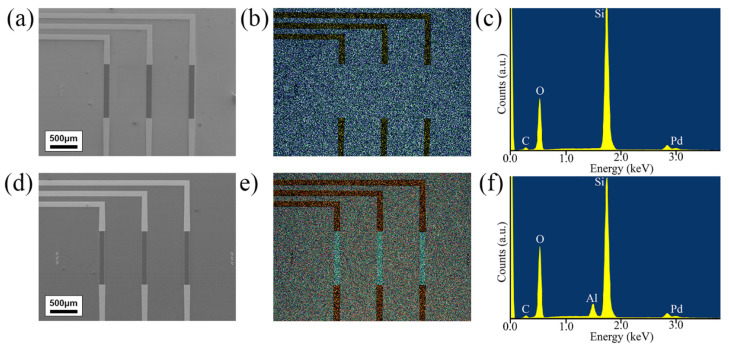
SEM and EDX images of non-passivated and passivated graphene devices. Colours used for EDX are blue (silicon), green (oxygen), yellow (palladium), and red (aluminium). (**a**) SEM image of a non-passivated device, with (**b**) EDX mapping image and (**c**) EDX spectrum. (**d**) SEM image of a passivated device, with (**e**) EDX mapping image and (**f**) EDX spectrum.

SEM images were performed on the non-passivated (Figure 4a) and passivated (Figure 4d) graphene devices to show the passivation across the graphene channel, with EDX mapping images displaying the elemental composition layout (Figure 4b,e) for each SEM image. The non-passivated devices spectrum show peaks for carbon Kα at 0.27 eV, oxygen Kα at 0.53 eV, silicon Kα at 1.75 eV [61], and the largest palladium Lα peak at 2.85 eV [62]. Once passivated, there is an additional peak at 1.47 eV for the aluminium [63] and the intensity of the oxygen peak increases due to the additional oxygen found in the Al_2_O_3_ passivation coating. These EDX results indicate that the Al_2_O_3_ MVD process has coated the entire graphene device and that the wet etching process to create the windows for the exposed graphene was successful.

#### 3.2.2. Surface Morphology—Atomic Force Microscopy (AFM)

**Figure 5:**
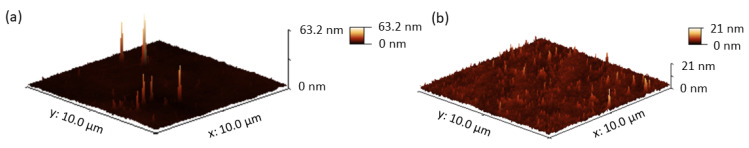
Representative surface morphology of the graphene channels, using AFM (**a**) on a non-passivated graphene device; and (**b**) a passivated graphene device, with Al_2_O_3_ deposited by MVD.

The surfaces of graphene channels in non-passivated and passivated devices were characterised for their topology using AFM. The surface roughness of the graphene channels in the non-passivated and passivated graphene devices (Figure 5) shows a clear change in the surface topography. The root mean square (RMS) roughness values were obtained from multiple locations on the surface. The RMS roughness of the non-passivated graphene device was 1.21 nm (Figure 5a). After the passivation procedure, the RMS roughness decreases to 0.89 nm (Figure 5b). Additionally, the size distribution of the photoresist residues present on the graphene surface substantially decreases, with a significant decrease of the larger globules of resist after passivation.

#### 3.2.3. Surface Chemistry Characterisation—Raman Spectroscopy

**Figure 6:**
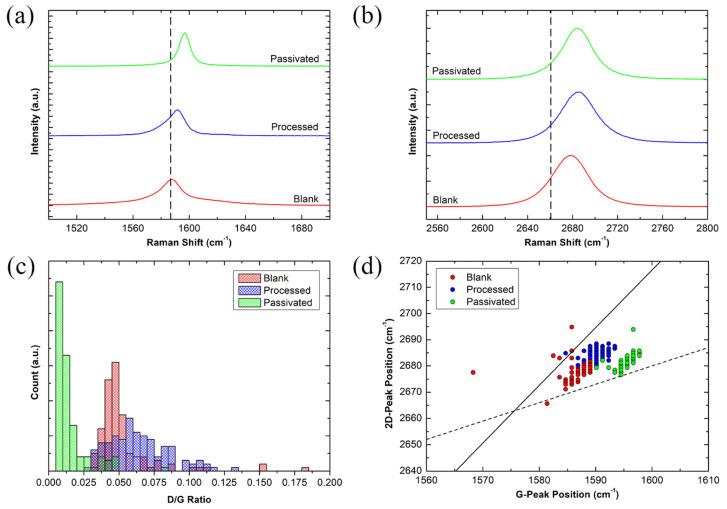
Raman spectroscopy of graphene before device processing (red), after device processing (blue), and after MVD Al_2_O_3_ deposition and etching the graphene window. (**a**) Raman spectra of the G-peak from 1500 to 1700 cm^−1^; and (**b**) the 2D-peak from 2550 to 2800 cm^−1^, (**c**) I_D_/I_G_ against count histogram; (**d**) and G- and 2D-peak position shift.

Raman spectroscopy was measured on unprocessed graphene (“Blank”); graphene after device fabrication at two stages, before Al_2_O_3_ MVD deposition (“Processed”) and after Al_2_O_3_ MVD passivation (“Passivated”). Figure 6a and b show the Raman spectra for the G-peak and 2D-peak, respectively, during each step of the graphene device fabrication process.

The ratio between the D-peak and G-peak intensities (I_D_/I_G_) can be used to quantify the ratio between sp^3^ and sp^2^ bonds, indicative of structural defects in graphene [64,65,66]. Figure 6c plots the I_D_/I_G_ of Blank, Processed, and Passivated graphene in a histogram. The I_D_/I_G_ of the blank graphene averages 0.057 with a large majority of measurements in the 0.03 to 0.06 range. These defects may be due to PMMA residue remaining on the surface after the graphene transfer. After graphene device fabrication, but before MVD deposition, the average I_D_/I_G_ increased to 0.062 with the range of many of the ratios being much larger, between 0.03 to 0.09. As explained in Figure 1, the device fabrication process requires patterning UV-sensitive photoresist to create both the graphene channels and the metal contacts. As a result, there can be residual photoresist residue present on the graphene surface after processing [67]. For biosensing purposes, this negatively impacts device fabrication consistency as it reduces the available graphene surface area and causes electron scattering, hindering the graphene electron mobility [68,69,70]. The presence of trace photoresist residue results in a wider range of I_D_/I_G_ values and a higher average I_D_/I_G_. Once the graphene is encapsulated with the MVD Al_2_O_3_ layer and the graphene windows have been etched out, the graphene is once again characterised using Raman spectroscopy. The number of defects significantly decreased with the I_D_/I_G_ being significantly lower, at 0.015. The range is also greatly reduced, with the majority of the I_D_/I_G_ readings being near the 0.0 to 0.02 range. The decrease in the I_D_/I_G_ means that a resist removal effect had taken place on the graphene surface, once it has been in contact with the Al_2_O_3_. In a publication by Do Van Lam et al., it has been reported that ALD Al_2_O_3_ has various types of “healing effects” in CVD graphene, such as resist residue, defects, and grain boundaries. These residue and defect areas provide nucleation sites for the ALD Al_2_O_3_ to grow on [40,49]. As the TMA/H_2_O cycles require binding to surface -OH functional groups, any surface contaminants providing those hydroxyl groups can be used by the MVD Al_2_O_3_ growth process [40]. After performing the TMAH etch, creating the graphene window in the passivation layer, any surface organic contaminants that were bound to the graphene surface may have been removed along with the Al_2_O_3_, which is consistent with a drop in the I_D_/I_G_.

As seen in Figure 6d, the presence of photoresist residue in noticeable blue shifts in the G- and 2D-peaks. It has been reported that the presence of charged impurities, such as photoresist, can lead to a change in the graphene charge distribution, affecting the Raman fingerprint and resulting in Raman peak shifts [71]. It is known that graphene on SiO_2_ dielectric is heavily p-doped [72] and the presence of the trace photoresist, after graphene device fabrication, adds additional strain on the graphene, seen by the shift in its Raman spectrum. With the presence of the Al_2_O_3_ on the graphene surface, this stress is relieved and an inherent n-doping effect takes place, causing a redshift in the Raman peaks (Figure 6d) [71,73,74,75,76].

#### 3.2.4. Surface Chemistry Characterisation—X-ray Photoelectron Spectroscopy (XPS)

**Figure 7:**
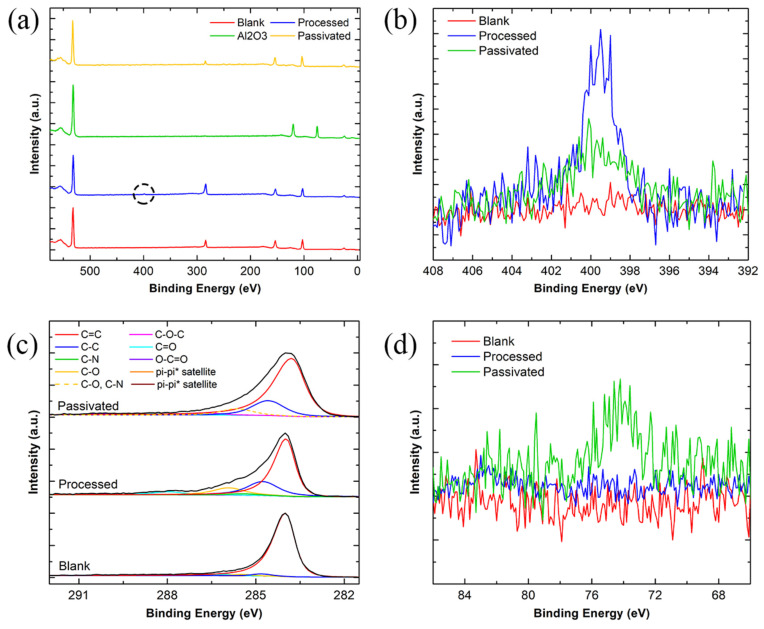
XPS spectra of blank (red), processed (blue), deposited Al_2_O_3_ (green), and passivated (yellow) graphene devices. (**a**) Wide spectra showing the O 1s, C 1s, Si 2p, and Al 2p peaks, with a black circle showing the small N 1s peak present in the “Processed” spectra; (**b**) zoomed-in view of the N 1s peak between the three different spectra; (**c**) C 1s spectra of blank, processed, and passivated graphene, showing the changes in sp^2^ carbon during each process step; and (**d**) Al 2p spectra showing that there is still a trace of Al_2_O_3_ present.

The presence of sp^3^-bonded carbon and other non-carbon elements on the graphene surface signifies the presence of defects and photoresist residue contamination, as pristine graphene is purely sp^2^ carbon. Wide spectra of the graphene were measured at each major step of the fabrication process, as shown in Figure 7a, showing the O 1s, C 1s, and Si 2p peaks at ~532, ~284, and ~103 eV [77]. A wide spectrum of the Al_2_O_3_ deposition, before etching, is also shown with Al 2p peaks at ~75 eV [77] and the surface was cleaned using an Ar GCIS. The wide spectra do not show the nitrogen peak due to how little nitrogen is present on the graphene surface except for a small visible peak in the “Processed” spectra, highlighted with a black circle. The nitrogen peak, at ~400 eV, is present in the XPS spectra due to the polyaliphatic imide copolymer found in the LOR 3A photoresist, making up approximately 1–20% of the photoresist (Figure 7b) [77,78,79]. After passivation, this nitrogen peak is reduced by 50%, indicating that the Al_2_O_3_ bound itself to the photoresist during the MVD growth stage and the photoresist was subsequently removed along with the Al_2_O_3_ during the wet etching stage. The atomic concentration of all the elements found in each spectrum are displayed in Table 1. 

The carbon 1s spectra for blank, processed, and passivated devices are displayed in Figure 7c. There is a very minimal photoresist residue remaining on the blank graphene surface from the PMMA-based wet transfer process. This is suggested by the large sp^2^ carbon peak at 284 eV, which represents the C=C component in graphene [80], whilst the additional peaks representative of sp^3^ carbon, C-O, and C=O at 284.79, 285.48, and 286.12 eV, respectively, are minimal, with the calculated area of sp^2^ carbon representing 94.44% of the total area of the spectra [30,81]. However, after processing the graphene into graphene devices, the presence of C-C sp^3^, C-O, and C=O peaks increase, with the formation of additional C-N [27,82], C-O-C, and O-C=O bonds at 285.42, 286.73, and 290.35 eV [30,81], respectively. The peak area of sp^2^ carbon also greatly decreases, only amounting to 57.16% of the total area of the carbon spectra. The presence of the C-N bond is due to the previously mentioned imide groups present on the graphene surface. Photoresists contain lots of C=O and C-OH molecules, as they are made up of resins and epoxies. The TMA precursor can bind to these C-O and C=O bonds, forming Al_2_O_3_ on top of the photoresist [40,83]. Al_2_O_3_ can also bind to defects present in the graphene hexagonal lattice, due to the previously discussed “healing effect” [49]. As the Al_2_O_3_ is removed, organic contaminants, such as photoresist, that were present on the graphene are also removed. However, the photoresist may not be removed completely, as indicated by the persistent photoresist-related peaks, such as the C-O, C-O-C, and C=O peaks. There is a noticeable increase of sp^2^ carbon peak area, compared to the “Processed” spectra. As previously mentioned, sp^2^ carbon only covers 57.16% of the total area in the “Processed” spectra and the area of sp^2^ carbon covers 70.17% of the total area of the Passivated” spectra, suggesting that the presence of the photoresist residue has decreased. Additionally, comparing the ratio of carbon to nitrogen in Table 1 between the “Processed” and “Passivated” results, suggests that there is a decreased presence of nitrogen-based compounds, normally found in photoresist, on the graphene surface. Figure 7d shows the trace aluminium present on the graphene surface after etching. This is largely due to traces of Al_2_O_3_ binding to the graphene defects and grain boundaries. The binding of Al_2_O_3_ chemically electron dopes the graphene, improving the graphene electron carrier density [49,84]. As seen in Figure 6c, the Al_2_O_3_-graphene binding improves the graphene material quality, reducing the number of defects present, and improving performance.

#### 3.2.5. The pDAN Electrochemical Functionalisation

Certain functionalisation processes require the utilisation of aggressive chemicals, such as sulphuric acid, for pDAN functionalisation, and a hydrogen peroxide and iron (II) sulphate mixture, for APTES functionalisation [14,30]. If left unprotected, these chemicals can oxidise the metal contacts which can begin to delaminate, resulting in the loss of electrical contact with the graphene. The electropolymerisation of DAN creates a polymer film across the graphene surface, which introduces -NH_2_ functional groups on the graphene surface, allowing for antibodies to bind to the amine groups for sensing purposes. It has been reported that pDAN is very robust, capable of remaining on the graphene surfaces after multiple subsequent washes due to strong Van der walls forces [27]. The electrochemical polymerisation process of polymerising DAN through CV [30,85] uses dilute H_2_SO_4_ as the electrolyte for covalently binding the monomers.

**Figure 8:**
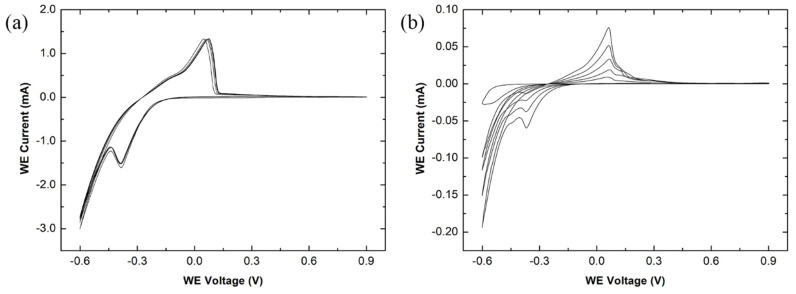
Cyclic voltammetry curves of electropolymerising 10 mM 1,5-diaminonaphthalene with 0.25 M H_2_SO_4_ for five cycles, with a working electrode voltage ranging from −0.6 to 0.9 V, and a scan rate of 50 mV/s. The electropolymerisation was performed on (**a**) non-passivated and (**b**) Al_2_O_3_-passivated graphene devices.

Moreover, 10 mM of DAN was mixed via sonication with 0.25 M H_2_SO_4_ solution, diluted down from 95–98% (~18 M) concentrated H_2_SO_4_, and was electropolymerised onto both the non-passivated and passivated CVD graphene devices at a scan rate of 50 mV/s, between −0.6 and 0.9 V, for five cycles, with the CV graphs shown in Figure 8. Five CV cycles were chosen to produce a thin layer of pDAN across the graphene surface, chemically modifying the graphene with -NH_2_ functional groups for further bio-functionalisation. The position of the oxidation and reduction peaks during electropolymerisation are similar between the non-passivated and passivated CV graphs, located at 0.075 and −0.39 V, 0.071 and −0.38 V, respectively. A small, broad oxidation peak is present at 0.32 V for the passivated graphene device, which represents the irreversible oxidation of the DAN monomers during the polymerisation step [86].

**Figure 9:**
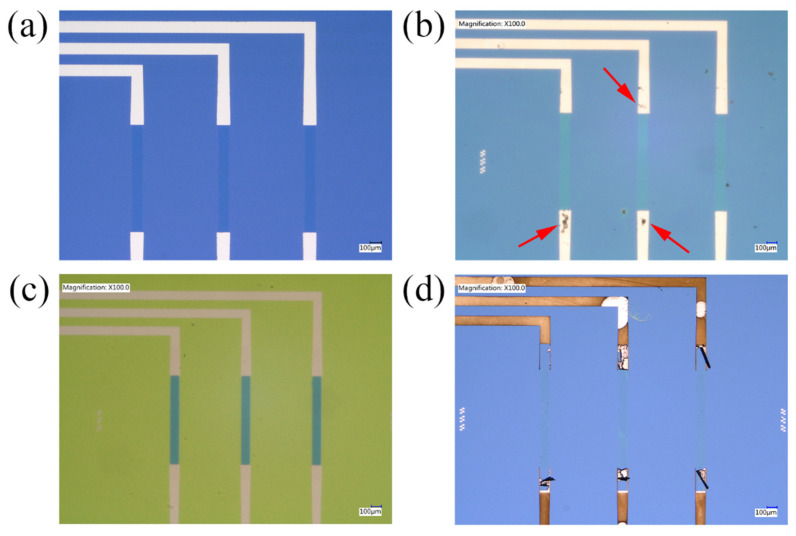
Optical microscope images of graphene devices before and after pDAN functionalisation. (**a**) A zoomed-in optical microscope image of a non-passivated graphene device before pDAN electropolymerisation. A zoomed-in optical microscope image of (**b**) a non-passivated device and (**c**) an Al_2_O_3_-passivated device, that has undergone five cycle pDAN electropolymerisation using the DAN/H_2_SO_4_ solution. (**d**) A zoomed-in optical microscope image of a non-passivated graphene device that underwent a 20 cycle pDAN electropolymerisation process.

It has been reported that the increase in current density, across the working electrode, after each cycle is evident that the DAN is polymerising on top of the CVD graphene [30]. This is evident in the passivated graphene devices CV scans, where the oxidation peak current initially begins at 8.92 μA before increasing to 75.71 μA after the fifth cycle. However, the oxidation and reduction peak positions and currents are similar across each cycle for the non-passivated graphene devices, with only small changes occurring. The graphene is still being polymerised, as evident by the changes in the colour of the graphene channels in Figure 9b. Additionally, the height of the anodic and cathodic peaks currents is greater in the non-passivated device compared to the passivated device. A significant portion of the current is a result of the highly conductive metal tracks contacting the DAN/H_2_SO_4_ solution, increasing the electron transfer in the electrochemical reaction [87]. The cathodic and anodic peak currents for the non-passivated and passivated graphene devices are 1.34 and −3.00 mA, 0.076 and −0.19 mA, respectively. 

After five cycles of electropolymerisation, the non-passivated graphene devices developed damage along the metal tracks which can be seen under an optical microscope. Areas of peeling metal are visible and are marked with red arrows in Figure 9b. For the passivated devices, the metal tracks are completely intact, with no visible degradation, signifying that the passivation process successfully protected the graphene (Figure 9c). These are compared to a non-passivated graphene device’s original state before passivation and functionalisation (Figure 9a). To determine the extent of potential damage to metal, a non-passivated graphene device underwent a 20 CV cycle electropolymerisation process to imitate the need for a thicker pDAN layer for biosensing purposes [30,88]. Seen in Figure 9d, after 20 electropolymerisation cycles, the metal tracks were completely oxidised and have delaminated from the surface. The metal tracks are no longer in contact with the graphene, disconnecting the graphene from the rest of the device.

## 4. Conclusions

We have demonstrated a fabrication process to improve the reliability of graphene biosensing devices using a novel MVD dielectric deposition passivation layer for graphene sensor devices, protecting the metal electrodes from aggressive chemicals. The process aids in cleaning and restoring the graphene surface, removing residual resist, and improving reproducibility and device consistency, an essential key development aspect for graphene-based biosensors. Raman analysis demonstrated an increase in a large range of defects present on the graphene surface after graphene lithographic device processing, which was subsequently greatly reduced and homogenised after Al_2_O_3_ deposition, rejuvenating the graphene. This can be seen from the XPS data, where the sp^2^ carbon component increases from 57.16 to 70.17%, showing a decrease in non-graphitic carbon present on the graphene surface, and a decrease in the nitrogen concentration of 50%, associated with reduced quantities of surface residue resist. A reliable Al_2_O_3_ wet etching process has been created and optimised, capable of patterning Al_2_O_3_ with a photoresist mask without negatively affecting the graphene substrate or the integrity of the Al_2_O_3_ layer. This improvement in graphene device consistency and the effective protection, that the Al_2_O_3_ passivation layer provides, lays the foundation for much more reliable graphene biosensing platforms, which can be integrated for commercial applications and point-of-care testing.

## Figures and Tables

**Table 1:** Atomic concentrations of carbon, oxygen, silicon, nitrogen, and aluminium present on the graphene surface for each graphene device fabrication process step, unprocessed (“Blank”), before MVD deposition (“Processed”) and after creating the MVD window (“Passivated”), obtained through X-ray photoelectron spectroscopy.

Sample	Carbon (%)	Oxygen (%)	Silicon (%)	Nitrogen (%)	Aluminium (%)
Blank	23.94 ± 0.82%	49.40 ± 0.70%	26.65 ± 0.64%	N/A	N/A
Processed	38.47 ± 1.23%	39.13 ± 1.34%	21.03 ± 0.96%	1.38 ± 1.17%	N/A
Passivated	19.33 ± 1.12%	52.54 ± 0.76%	26.94 ± 0.61%	0.65 ± 0.88%	0.54 ± 0.34%

## Data Availability

Data available upon request.
